# Prevalence and determinants of non-alcoholic fatty liver disease in lifelines: A large Dutch population cohort

**DOI:** 10.1371/journal.pone.0171502

**Published:** 2017-02-02

**Authors:** Eline H. van den Berg, Marzyeh Amini, Tim C. M. A. Schreuder, Robin P. F. Dullaart, Klaas Nico Faber, Behrooz Z. Alizadeh, Hans Blokzijl

**Affiliations:** 1 Department of Gastroenterology and Hepatology, University Medical Center Groningen, University of Groningen, Groningen, The Netherlands; 2 Department of Epidemiology, University Medical Center Groningen, University of Groningen, Groningen, The Netherlands; 3 Department of Endocrinology, University Medical Center Groningen, University of Groningen, Groningen, The Netherlands; Università degli Studi di Palermo, ITALY

## Abstract

**Background & aims:**

Non-alcoholic fatty liver disease is an increasing health issue that develops rather unnoticed with obesity, type 2 diabetes mellitus and metabolic syndrome. We investigated prevalence, determinants and associated metabolic abnormalities of non-alcoholic fatty liver disease in the largest population-based cohort to date.

**Methods:**

Biochemical characteristics, type 2 diabetes mellitus and metabolic syndrome were determined in the Lifelines Cohort Study (N = 167,729), a population-based cohort in the North of the Netherlands. Non-alcoholic fatty liver disease was defined as Fatty Liver Index (FLI)≥60. Exclusion criteria were age <18 years, immigrants, missing data to assess FLI and metabolic syndrome, excessive alcohol use, previous-diagnosed hepatitis or cirrhosis and non-fasting blood sampling.

**Results:**

Out of 37,496 included participants (median age 44 years, 62.1% female), 8,259 (22.0%) had a FLI≥60. Individuals with a FLI≥60 were more often male, older, obese, had higher levels of hemoglobinA1c, fasting glucose, liver enzymes, total cholesterol, low-density lipoprotein cholesterol, triglycerides, c-reactive protein and leucocytes and lower high-density lipoprotein cholesterol (all *P*<0.0001). Participants with a FLI≥60 showed higher prevalence of type 2 diabetes mellitus (9.3% vs. 1.4%), metabolic syndrome (54.2% vs. 6.2%), impaired renal function (20.1% vs. 8.7%) and cardiovascular disease (4.6% vs. 1.6%) (all *P*<0.0001). Multivariable logistic analysis showed that smoking, hemoglobin, leucocytes, c-reactive protein, platelets, alanine aminotransferase, alkaline phosphatase, albumin, impaired renal function (OR 1.27, 95%CI 1.15–1.41), metabolic syndrome (OR 11.89, 95%CI 11.03–12.82) and its individual components hyperglycemia (OR 2.53, 95%CI 2.34–2.72), hypertension (OR 1.89, 95%CI 1.77–2.01) and reduced high-density lipoprotein cholesterol (OR 3.44, 95%CI 3.22–3.68) were independently associated with suspected non-alcoholic fatty liver disease (all *P*<0.0001).

**Conclusion:**

Twenty-two percent (22.0%) of the population in the North of the Netherlands is suspected to suffer from non-alcoholic fatty liver disease, coinciding with a significant increased risk of type 2 diabetes mellitus, metabolic syndrome, cardiovascular disease and impaired renal function.

## Introduction

Non-alcoholic fatty liver disease (NAFLD) is characterized by hepatic steatosis in the absence of excessive alcohol consumption. The spectrum of NAFLD ranges from simple steatosis to non-alcoholic steatohepatitis (NASH), fibrosis and ultimately cirrhosis with its known complications, such as decompensation and hepatocellular carcinoma (HCC)[1]. In patients with NASH, progression to fibrosis occurs in 40.8% with a liver specific mortality hazard ratio of 2.6[2]. As a result of the global obesity epidemic, NAFLD is an increasing relevant public health issue and emerging as the most common cause of chronic liver disease in Western countries. It is expected to become the most important indication for liver transplantation in the near future[3]. Although most patients with NAFLD are not at risk of dying from liver disease, they have a substantial increased risk of early morbidity and mortality[1,4,5]. NAFLD frequently co-exists with metabolic disorders and the association with the metabolic syndrome (MetS) is strong[6]. Another condition associated with NAFLD is cardiovascular disease, with increased intima-media thickness and carotid plaques representing progressive atherosclerosis[7].

In European countries the prevalence of NAFLD has been reported to range widely from 3.1–41.2% (S1 Table), and is likely to increase over the coming years[8–24]. Nonetheless, there are only few, mostly small-sized European epidemiological studies analyzing the prevalence of NAFLD in the general population[8,9,13,20], with the largest survey being performed in only 4,222 participants[20]. Other studies were performed in selected categories adapted from a general population[10–12,14–19,21–24].

Given considerable variation in reported prevalence numbers derived from rather small cohorts, and the increasing incidence of NAFLD with its serious consecutive complications and comorbidity, the present study was initiated to establish a comprehensive sufficiently powered analysis on the prevalence of NAFLD. Here, we aimed to investigate the prevalence, determinants and comorbid conditions of NAFLD in a large population-based cohort from the North of the Netherlands.

## Methods

### Study design

This cross-sectional study was conducted within the framework of the Lifelines Cohort Study[25–27]. The Lifelines Cohort Study is a multi-disciplinary prospective population-based cohort study of 167,729 persons living in the North of the Netherlands. It employs a broad range of investigative procedures in assessing the biomedical, socio-demographic, behavioral, physical and psychological factors which contribute to the health and disease of the general population, with a special focus on multi-morbidity and complex genetics. Participants were recruited via general practitioners, subsequently family members were invited to participate and finally, adults could self-register to participate. All participants provided written informed consent. The medical ethics committee of the University of Groningen, the Netherlands, approved the study[25–27].

### Study participants

Subjects of Western-European origin were included. All study participants were aged between 18–91 years at time of enrollment. Exclusion criteria were participants <18 years, those with missing data required to calculate the Fatty Liver Index (FLI)[28] (described below) and to determine MetS components, non-fasting participants at time of blood collection, immigrants, participants with self-reported excessive alcohol use and those previous diagnosed with hepatitis or cirrhosis. Information about nationality, fasting state, smoking, medication use, alcohol consumption, hepatitis B virus infection and cirrhosis was extracted from the self-administered questionnaires. Participants were assumed to be of Western-European origin if his/her birth country and that of both parents was the Netherlands, which is in accordance with the definition of Statistics of the Netherlands[27]. Participants were considered normal drinkers when daily alcoholic intake was ≤1 drink in females and ≤2 drinks in males[29]. Current smokers consisted of participants with active smoking or smoking in the past month.

### Data collection and measurements

Data was collected in the Lifelines Cohort Study between 2006–2013. Questionnaires were collected, anthropometry and blood pressure were measured and biomaterial (blood) was collected at the Lifelines research sites. A standardized protocol was used to obtain blood pressure and anthropometric measurements (height, weight and waist circumference). Systolic and diastolic blood pressures were measured 10 times during a period of 10 minutes, using an automated Dinamap Monitor (GE Healthcare, Freiburg, Germany). The size of the cuff was chosen according to the arm circumference. The average of the final three readings was used for each blood pressure parameter. Anthropometric measurements were measured without shoes. Body weight was measured to the nearest 0.5 kg. Height and waist circumference were measured to the nearest 0.5 cm. Height was measured with a stadiometer placing their heels against the rod and the head in Frankfort Plane position. Waist circumference was measured in standing position with a tape measure all around the body at the level midway between the lower rib margin and the iliac crest[25,26].

Venous blood samples were collected between 8.00–10.00 a.m. into heparin-containing tubes, centrifuged at 1,885x*g* and the plasma aliquots were processed for laboratory measurements at the same day and stored at -80°C. Hemoglobin, total leucocytes and platelets were measured using routine procedures on a XE2100-system (Sysmex, Japan). High-sensitivity c-reactive protein (CRP) was measured with CardioPhase hs CRP (Siemens, BNII, Germany) and from 2012 with CRPL3 on a Roche Modular P chemistry analyzer. Total cholesterol, low-density lipoprotein (LDL) cholesterol, high-density lipoprotein (HDL) cholesterol and triglycerides (TG) were measured using routine procedures on a Roche Modular P chemistry analyzer. Glucose was assayed with the UV-test hexokinase method on a Roche Modular P chemistry analyzer and hemoglobin A1c (HbA1c) was measured with high performance liquid chromatography (HPLC) (Roche). Gamma-glutamyltransferase (GGT), alkaline phosphatase (ALP), alanine aminotransferase (ALT) and aspartate aminotransferase (AST) were quantified according to the recommendation of the International Federation of Clinical Chemistry on a Roche Modular Platform. ALT and AST were measured with pyridoxal phosphate activation. Albumin was measured with a BCG albumin assay kit for colorimetric testing on a Roche Modular P chemistry analyzer. All laboratory measurements were performed with standardized laboratory measurements and quality assessment control at the Department of Laboratory Medicine of the University Medical Center Groningen, the Netherlands[25,26].

### Definition of NAFLD

For the diagnosis of NAFLD the algorithm of the Fatty Liver Index (FLI) was used. The FLI was calculated according to the formula published by Bedogni[28]. *FLI = (e*^*0*.953^*^loge (triglycerides +0.139^*^BMI+0.718^*^loge (GGT)+0.053^*^waist circumference–15.*745*^*)/(1+e*^*0*.953^*^loge (triglycerides)+0.139^*^BM +0.718^*^loge(GGT +0.053^*^waist circumference–15.*745*^)*100, where GGT is gamma-glutamyltransferase. The optimal cut-off value for the FLI has been documented to be 60 with an accuracy of 0.84, a sensitivity of 61% and a specificity of 86% for detecting NAFLD as determined by ultrasonography[28]. A FLI≥60 was thus used as a proxy of NAFLD. The 2016 EASL-EASD-EASO NAFLD guideline recommends that for larger scale screening studies, serum biomarkers are the preferred diagnostic tool with the FLI currently considered to be one of the best validated steatosis scores[29].

### Definition of comorbid diseases

Computational models for the determination of comorbid diseases were used. For the definition of obesity the body mass index (BMI) was used, calculated as weight (kg) divided by height squared (m^2^). The diagnosis of type 2 diabetes mellitus (T2DM) was confirmed when a subject had either self-reported on T2DM, used glucose lowering medication, had a fasting glucose (FG) ≥7.0 mmol/L or a HbA1c ≥47.5 mmol/mol. MetS was defined by the revised diagnostic criteria from the American Heart Association by the National Cholesterol Education Program Adult Treatment Panel III[30] and consist of five criteria: *(1)* enlarged waist circumference (males ≥102 cm and females ≥88 cm), *(2)* elevated TG (≥1.7 mmol/L) and/or medication use for elevated TG, *(3)* reduced HDL cholesterol (males <1.0 mmol/L and females <1.3 mmol/L) and/or medication use for reduced HDL cholesterol, *(4)* elevated blood pressure (systolic blood pressure ≥130 mmHg or diastolic blood pressure ≥85 mmHg) and/or medication use for hypertension, *(5)* elevated fasting glucose (≥5.6 mmol/L) and/or medication use for elevated glucose. Participants were diagnosed with MetS when at least three out of five criteria were present[30]. The presence of a self-reported history of myocardial infarction, percutaneous coronary intervention, coronary artery bypass surgery, stroke or the diagnosis of narrowing of one or both carotid arteries was defined as atherosclerotic cardiovascular disease. Chronic impaired renal function was defined by calculating the estimated glomerular filtration rate (eGFR) < 60 ml/min/1.73m^2^, using the Modification of Diet in Renal Disease (MDRD) Study Equation [31,32].

### Data analyses and statistical modeling

Statistical analyses was performed with SPSS (version 22.0, SPSS Inc., Chicago, IL, USA). Data are expressed in means with standard deviations (SD), medians with interquartile ranges (IQR) and in numbers with percentages. Normality of distribution was assessed and checked for skewness. Variables were compared between FLI≥60 and FLI<60 groups using Student T-test, Mann-Whitney U test Chi-square test. To preclude interactions with the dependent factor FLI≥60, all variables in the equation defining the FLI (i.e. BMI, waist circumference, TG and GGT) were excluded in multivariable analyses. Due to correlations ≥0.5; AST (correlation with ALT), glucose (correlation with HbA1c) and total cholesterol (correlation with LDL cholesterol) were excluded from multivariable analyses and residual variables were made to exclude remaining interactions. For continuous variables a Z-score was calculated and used in multivariable analyses. Stepwise binary logistic regression analyses was performed to disclose the independent association of a FLI≥60. Results are presented by odds ratio (OR) with 95% confidence intervals (CI). To account for the number of independent tests, we applied a Bonferroni correction. Two-sided *P*-values of <0.001 (0.05/60) were considered statistically significant, given the use of 60 independent tests embedded in 4 multivariable models.

## Results

**Fig 1:**
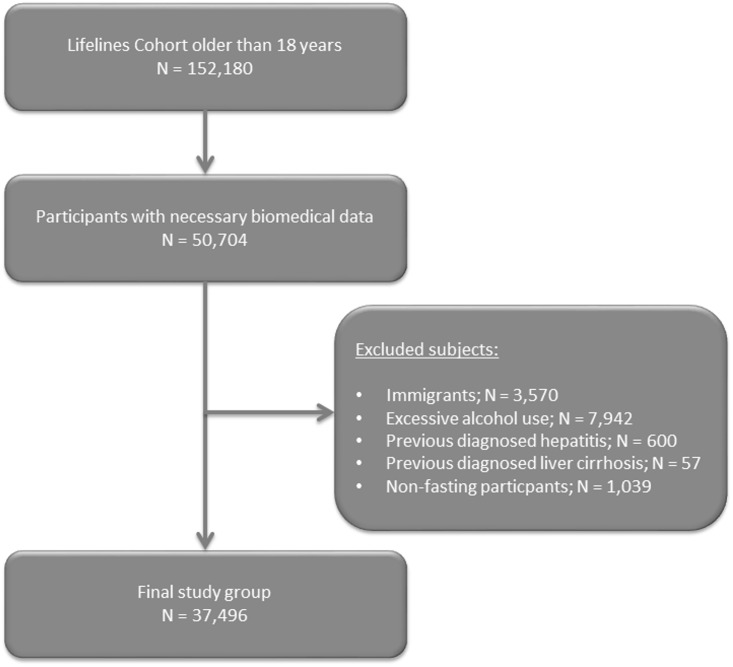
Flow chart of the study population.

From the 167,729 participants of the Lifelines Cohort Study, 152,180 participants were older than 18 years and 50,704 participants were eligible for our study with necessary available biomedical data concerning the calculation for the FLI and MetS. After applying exclusion criteria, the final study group consisted of 37,496 participants (Fig 1). The median age of the study group was 44 years, with a median BMI of 25.5 kg/m^2^ and was predominantly female (62.1%). Population characteristics are presented in Table 1.

**Table 1:** Population characteristics.

***Baseline characteristics***	**N = 37,496**
Sex: females, *n (%)*	23,270 (62.1)
Age (years), *median (IQR)*	44 (36–51)
BMI (kg/m^2^), *median (IQR)*	25.5 (23.1–28.4)
BMI	
Normal; ≤ 25 kg/m^2^, *n (%)* Overweight; 25–30 kg/m^2^, *n (%)* Obese; ≥ 30 kg/m^2^, *n (%)*	16,649 (44.4) 14,696 (39.2) 6,151 (16.4)
Waist circumference (cm)	
Male, *median (IQR)* Female, *median (IQR)*	95 (88–102) 86 (78–95)
Smoking, *n (%)*	7,008 (18.9)
***Blood tests***	
Hemoglobin (mmol/L), *median (IQR)*	8.6 (8.1–9.2)
ALT (U/L), *median (IQR)*	19 (14–27)
AST (U/L), *median (IQR)*	22 (19–27)
GGT (U/L), *median (IQR)*	19 (14–28)
ALP (U/L), *mean ± SD*	62 ± 18
Albumin (g/L), *mean ± SD*	44.9 ± 2.4
Platelets (×10^9^/L), *mean ± SD*	249.9 ± 56.8
HbA1c (mmol/mol), *median (IQR)*	38.0 (35.0–40.0)
Fasting glucose (mmol/L), *median (IQR)*	4.9 (4.6–5.2)
HDL cholesterol (mmol/L), *median (IQR)*	1.4 (1.2–1.7)
LDL cholesterol (mmol/L), *median (IQR)*	3.1 (2.5–3.7)
Triglycerides (mmol/L), *median (IQR)*	1.0 (0.7–1.4)
Total cholesterol (mmol/L), *median (IQR)*	4.9 (4.3–5.6)
CRP (mg/L), *median (IQR)*	1.2 (0.6–2.9)
Total leucocytes (×10^9^/L), *median (IQR)*	5.8 (4.9–6.9)
***Comorbidities***	
Type 2 diabetes mellitus, *n (%)*	1,199 (3.2)
Metabolic syndrome, *n (%)*	6,346 (16.9)
Abdominal obesity, *n (%)*	14,110 (37.6)
Hyperglycemia, *n (%)*	4,858 (13.0)
Hypertension, *n (%)*	14,021 (37.4)
Elevated TG, *n (%)*	5,548 (14.8)
Low HDL cholesterol, *n (%)*	9,857 (26.3)
Cardiovascular disease, *n (%)*	840 (2.2)
Impaired renal function, *n (%)*	4,240 (11.3)

Data are given in number with percentages (%), mean ± standard deviation (SD) for normally distributed data or median with interquartile ranges (IQR) for non-normally distributed data. Metabolic syndrome was defined according to NCEP ATPIII criteria. Abbreviations: **ALP**, alkaline phosphatase; **ALT**, alanine aminotransferase; **AST**, aspartate aminotransferase; **BMI**, body mass index; **CRP**, c-reactive protein; **GGT**, gamma-glutamyltransferase; **HbA1c**, hemoglobin A1c; **HDL**, high-density lipoprotein; **LDL**, low-density lipoprotein; **TG**, triglycerides.

Suspected NAFLD was defined by FLI≥60. Suspected NAFLD was observed in 22.0% (8,259 participants) of the study group. Table 2 shows the clinical and laboratory characteristics in subjects with and without suspected NAFLD (FLI<60). Those with suspected NAFLD were older (median age 47 years) and more likely to be male; corresponding prevalence numbers were 32.7% in all males and 15.7% in all females, respectively. As expected, in the group with suspected NAFLD, more obese participants were detected (median BMI of 30.8 kg/m^2^) compared to those with a FLI<60 (median BMI of 24.4 kg/m^2^). T2DM (9.3% vs. 1.4%, *P*<0.0001) and MetS (54.2% vs. 6.2%, *P*<0.0001) were more prevalent in subjects with a FLI≥60. Significant differences for each individual MetS component were also present (all *P*<0.0001). Cardiovascular disease (4.6% vs. 1.6%, *P*<0.0001) and impaired renal function (20.1% vs. 8.7%, *P*<0.0001) were also more prevalent in subjects with a FLI≥60. In subjects with a FLI≥60, hemoglobin, total leucocytes, CRP, platelets, ALT, AST, GGT, ALP, HbA1c, FG, LDL cholesterol, TG and total cholesterol values were significantly higher and HDL cholesterol and albumin were lower. After adjusting for age and sex, these differences remained significant (all *P*<0.001).

**Table 2:** Clinical and laboratory characteristics in subjects with and without non-alcoholic fatty liver disease estimated by the Fatty Liver Index (FLI ≥ 60).

	FLI < 60 (N = 29,008)	FLI ≥ 60 (N = 8,259)	*P-value*	*P-value adjusted for age and sex*
***Baseline characteristics***				
Sex: females, *n (%)*	19,519 (67.3)	3,644 (44.1)	<0.0001	
Age (years), *median (IQR)*	43 (35–50)	47 (40–56)	<0.0001	
BMI (kg/m^2^), *median (IQR)*	24.4 (22.6–26.5)	30.8 (28.6–33.9)	<0.0001	<0.0001
BMI				
Normal; ≤ 25 kg/m^2^, *n (%)* Overweight; 25–30 kg/m^2^, *n (%)* Obese; ≥ 30 kg/m^2^, *n (%)*	16,514 (56.9) 11,377 (39.2) 1,117 (3.9)	127 (1.5) 3,161 (38.3) 4,971 (60.2)	<0.0001 0.12 <0.0001	<0.0001 <0.0001 <0.0001
Waist circumference (cm)				
Male, *median (IQR)* Female, *median (IQR)*	91 (85–96) 84 (77–90)	105 (100–111) 106 (100–112)	<0.0001 <0.0001	<0.0001
Smoking, *n (%)*	5,267 (18.3)	1,698 (20.7)	<0.0001	<0.0001
***Blood tests***				
Hemoglobin (mmol/L), *median (IQR)*	8.5 (8.1–9.1)	9.0 (8.5–9.6)	<0.0001	<0.0001
ALT (U/L), *median (IQR)*	17 (13–24)	27 (20–39)	<0.0001	<0.0001
AST (U/L), *median (IQR)*	22 (19–26)	25 (21–30)	<0.0001	<0.0001
GGT (U/L), *median (IQR)*	17 (14–24)	32 (23–47)	<0.0001	<0.0001
ALP (U/L), *mean ± SD*	60 ± 17	70 ± 20	<0.0001	<0.0001
Albumin (g/L), *mean ± SD*	45.0 ± 2.4	44.6 ± 2.4	<0.0001	<0.0001
Platelets (×10^9^/L), *mean ± SD*	249.3 ± 56.4	252.1 ± 58.2	<0.0001	<0.0001
HbA1c (mmol/mol), *median (IQR)*	37.0 (35.0–39.0)	39.0 (37.0–42.0)	<0.0001	<0.0001
Fasting glucose (mmol/L), *median (IQR)*	4.8 (4.6–5.1)	5.2 (4.9–5.7)	<0.0001	<0.0001
HDL cholesterol (mmol/L), *median (IQR)*	1.5 (1.3–1.7)	1.2 (1.0–1.4)	<0.0001	<0.0001
LDL cholesterol (mmol/L), *median (IQR)*	3.0 (2.5–3.6)	3.4 (2.8–4.1)	<0.0001	<0.0001
Triglycerides (mmol/L), *median (IQR)*	0.9 (0.7–1.2)	1.6 (1.2–2.2)	<0.0001	<0.0001
Total cholesterol (mmol/L), *median (IQR)*	4.8 (4.3–5.5)	5.3 (4.6–5.9)	<0.0001	<0.0001
CRP (mg/L), *median (IQR)*	1.0 (0.5–2.3)	2.3 (1.1–5.0)	<0.0001	<0.0001
Total leucocytes (×10^9^/L), *median (IQR)*	5.6 (4.8–6.7)	6.3 (5.4–7.5)	<0.0001	<0.0001
***Comorbidities***				
Type 2 diabetes mellitus, *n (%)*	415 (1.4)	771 (9.3)	<0.0001	<0.0001
Metabolic syndrome, *n (%)*	1,801 (6.2)	4,469 (54.2)	<0.0001	<0.0001
Abdominal obesity, *n (%)*	7,219 (24.9)	6,746 (81.7)	<0.0001	<0.0001
Hyperglycemia, *n (%)*	2,246 (7.8)	2,562 (31.1)	<0.0001	<0.0001
Hypertension, *n (%)*	8,937 (30.8)	4,960 (60.1)	<0.0001	<0.0001
Elevated TG, *n (%)*	1,819 (6.3)	3,660 (44.3)	<0.0001	<0.0001
Low HDL cholesterol, *n (%)*	5,491 (18.9)	4,263 (51.6)	<0.0001	<0.0001
Cardiovascular disease, *n (%)*	459 (1.6)	373 (4.6)	<0.0001	<0.0001
Impaired renal function, *n (%)*	2,537 (8.7)	1,656 (20.1)	<0.0001	0.0004

Data are given in number with percentages (%), mean ± SD or median with interquartile ranges (IQR). For comparison between two groups, T-test (for normally distributed variables) and Mann-Whitney U test were used for continuous variables and for binary variables Chi square test were used. For age- and sex-adjusted P-values binary logistic regression was used. FLI = (e ^0.953^*^loge (triglycerides) + 0.139^*^BMI + 0.718^*^loge (GGT) + 0.053^*^waist circumference—15.745^) / (1 + e ^0.953^*^loge (triglycerides) + 0.139^*^BMI + 0.718^*^loge (GGT) + 0.053^*^waist circumference—15.745^) * 100. Metabolic syndrome was defined according to NCEP ATPIII criteria. Abbreviations: **ALP**, alkaline phosphatase; **ALT**, alanine aminotransferase; **AST**, aspartate aminotransferase; **BMI**, body mass index; **CRP**, c-reactive protein; **GGT**, gamma-glutamyltransferase; **HbA1c**, hemoglobin A1c; **HDL**, high-density lipoprotein; **LDL**, low-density lipoprotein; **TG**, triglycerides.

In order to disclose the independent associations of a FLI≥60 with clinical and biochemical characteristics subsequent stepwise multivariable logistic regression was performed (Tables 3 and 4). In age- and sex- adjusted analysis, impaired renal function, current smoking, hemoglobin, total leucocytes, CRP, platelets, ALT, ALP, albumin, HDL cholesterol and LDL cholesterol were all independent factors associated with a FLI≥60 (all *P*<0.01) (Table 3). Of note, HbA1c (OR 1.10, 95%CI 1.07–1.14, *P*<0.0001) and T2DM (OR 2.31, 95%CI 1.97–2.70, *P*<0.0001) were both independently associated with a FLI≥60 (Table 3; model 1 vs. model 2). In consecutive analysis, the presence of MetS and its individual components were included (Table 4). Waist circumference, HDL cholesterol, TG, HbA1c and T2DM were excluded to preclude interactions of variables accounted for the MetS components and concurrent presence in the equation of the FLI. After inclusion of MetS in the model, independent associations of a FLI≥60 were found with impaired renal function, smoking, hemoglobin, total leucocytes, CRP, platelets, ALT, ALP and albumin (all *P*<0.0001) (Table 4, model 1 and 2). LDL cholesterol and a cardiovascular disease history were only significantly associated with FLI when the individual components of MetS were added (*P*<0.01) (Table 4, model 2). Besides a very strong association of a FLI≥60 with the presence of MetS (OR 11.89, 95%CI 11.03–12.82, *P*<0.0001) (Table 4; model 1), a FLI≥60 was also independently associated with all of the remaining individual MetS components; hyperglycemia (OR 2.53, 95%CI 2.34–2.72, *P*<0.0001), hypertension (OR 1.89, 95%CI 1.77–2.01, *P*<0.0001) and low HDL cholesterol (OR 3.44, 95%CI 3.22–3.68, *P*<0.0001) (Table 4; model 2).

**Table 3:** Multivariable logistic regression analyses demonstrating independent associations of non-alcoholic fatty liver disease estimated by the Fatty Liver Index (FLI ≥ 60) with current smoking, HbA1c and type 2 diabetes mellitus.

	Model 1			Model 2		
	OR	95% CI	*P*-value	OR	95% CI	*P*-value
**Age (years)**	1.042	1.039–1.045	<0.0001	1.041	1.038–1.044	<0.0001
**Sex (male vs. female)**	1.120	1.019–1.231	0.018	1.109	1.009–1.219	0.031
**Hemoglobin (mmol/L)**	1.434	1.370–1.502	<0.0001	1.430	1.366–1.497	<0.0001
**ALT (U/L)**	2.101	2.019–2.185	<0.0001	2.073	1.993–2.157	<0.0001
**ALP (U/L)**	1.336	1.293–1.381	<0.0001	1.341	1.298–1.386	<0.0001
**Albumin (g/L)**	0.830	0.800–0.860	<0.0001	0.822	0.792–0.852	<0.0001
**Platelets (x10**^**9**^**/L)**	1.176	1.136–1.217	<0.0001	1.187	1.146–1.229	<0.0001
**HbA1c (mmol/mol)**	1.103	1.068–1.138	<0.0001			
**HDL cholesterol (mmol/L)**	0.242	0.229–0.255	<0.0001	0.249	0.236–0.263	<0.0001
**LDL cholesterol (mmol/L)**	0.524	0.500–0.549	<0.0001	0.534	0.509–0.559	<0.0001
**CRP (mg/L)**	1.329	1.285–1.374	<0.0001	1.323	1.279–1.368	<0.0001
**Total leucocytes (x10**^**9**^**/L)**	1.115	1.073–1.158	<0.0001	1.107	1.066–1.151	<0.0001
**Current smoking**	1.521	1.398–1.655	<0.0001	1.482	1.362–1.612	<0.0001
**Cardiovascular disease**	1.046	0.874–1.251	0.625	1.110	0.926–1.330	0.260
**Impaired renal function**	1.143	1.036–1.262	0.008	1.156	1.047–1.276	0.004
**T2DM**				2.306	1.972–2.697	<0.0001

OR: odds ratio. For continuous variables ORs are expressed per SD increase. Residual variables for ALT, HbA1c and LDL were used. Binary logistic regression analysis was used for all models. Model 1: includes HbA1c; model 2: includes persence of T2DM. FLI = (e ^0.953^*^loge (triglycerides) + 0.139^*^BMI + 0.718^*^loge (GGT) + 0.053^*^waist circumference—15.745^) / (1 + e ^0.953^*^loge (triglycerides) + 0.139^*^BMI + 0.718^*^loge (GGT) + 0.053^*^waist circumference—15.745^) * 100. Abbreviations: **ALP**, alkaline phosphatase; **ALT**, alanine aminotransferase; **CRP**, c-reactive protein; **HbA1c**, hemoglobin A1c; **HDL**, high-density lipoprotein; **LDL**, low-density lipoprotein; **T2DM**, type 2 diabetes mellitus.

**Table 4:** Multivariable logistic regression analyses demonstrating independent associations of non-alcoholic fatty liver disease estimated by the Fatty Liver Index (FLI ≥ 60) with the presence of current smoking, the metabolic syndrome and its individual components.

	Model 1			Model 2		
	OR	95% CI	*P*-value	OR	95% CI	*P*-value
**Age (years)**	1.012	1.009–1.015	<0.0001	1.017	1.014–1.020	<0.0001
**Sex (male vs. female)**	1.748	1.586–1.926	<0.0001	1.473	1.344–1.615	<0.0001
**Hemoglobin (mmol/L)**	1.420	1.354–1.489	<0.0001	1.437	1.374–1.503	<0.0001
**ALT (U/L)**	1.999	1.921–2.081	<0.0001	2.065	1.987–2.147	<0.0001
**ALP (U/L)**	1.347	1.303–1.394	<0.0001	1.339	1.297–1.383	<0.0001
**Albumin (g/L)**	0.740	0.713–0.768	<0.0001	0.757	0.730–0.784	<0.0001
**Platelets (x10**^**9**^**/L)**	1.123	1.083–1.164	<0.0001	1.131	1.093–1.170	<0.0001
**LDL cholesterol (mmol/L)**	0.974	0.942–1.007	0.119	0.883	0.854–0.912	<0.0001
**CRP (mg/L)**	1.328	1.284–1.374	<0.0001	1.334	1.291–1.379	<0.0001
**Total leucocytes (x10**^**9**^**/L)**	1.162	1.118–1.208	<0.0001	1.179	1.136–1.224	<0.0001
**Current smoking**	1.295	1.186–1.414	<0.0001	1.317	1.212–1.431	<0.0001
**Cardiovascular disease**	1.040	0.859–1.257	0.689	1.303	1.091–1.557	0.004
**Impaired renal function**	1.272	1.148–1.408	<0.0001	1.216	1.104–1.340	<0.0001
**Metabolic syndrome**	11.888	11.029–12.815	<0.0001			
**- Hyperglycemia**				2.533	2.340–2.724	<0.0001
**- Hypertension**				1.885	1.768–2.009	<0.0001
**- Low HDL cholesterol**				3.443	3.221–3.681	<0.0001

OR: odds ratio. For continuous variables ORs are expressed per SD increment. Residual variables for ALT and LDL were used. Binary logistic regression analysis was used for all models. FLI = (e ^0.953^*^loge (triglycerides) + 0.139^*^BMI + 0.718^*^loge (GGT) + 0.053^*^waist circumference—15.745^) / (1 + e ^0.953^*^loge (triglycerides) + 0.139^*^BMI + 0.718^*^loge (GGT) + 0.053^*^waist circumference—15.745^) * 100. Metabolic syndrome was defined according to NCEP ATPIII criteria. Abbreviations: **ALP**, alkaline phosphatase; **ALT**, alanine aminotransferase; **CRP**, c-reactive protein; **HDL**, high-density lipoprotein; **LDL**, low-density lipoprotein.

## Discussion

In this large population based cross-sectional study among almost 40,000 subjects from the Northern part of the Netherlands, the prevalence of NAFLD and its associated metabolic derangements were studied demonstrating that 22% of this adult Western-European population is suspected to suffer from NAFLD. These individuals were more likely to be men, older and suffering from hypertension, T2DM, MetS, cardiovascular disease and impaired renal function. Laboratory tests revealed significant increased glucose, ALT and ALP levels and decreased HDL cholesterol. Further, current smoking, higher levels of hemoglobin, CRP and total leucocytes count were also independently associated with suspected NAFLD.

Previous European studies that have investigated the prevalence of NAFLD in the general populations reported outcomes ranging from 17.9–29.9% (S1 Table)[8,9,13,20]. Gastaldeli *et al*. found a prevalence of 17.9% in 1,307 participants from 14 different European countries by the use of the FLI (FLI>60)[8]. Three other single country cohorts of the general populations from Germany[20], Spain[13] and Italy[9] demonstrated a NAFLD prevalence by ultrasonography of 29.9%[20], 25.8%[13] and 22.6%[9], respectively. However, these studies represented only 4,222[20], 766[13] and 598[9] participants. Other small European prevalence studies used specific categories of general populations introducing potential bias (e.g. hospitalized patients, heavy drinkers, obese subjects and deceased patients) (S1 Table)[10–12,14–19,21–24]. A recent meta-analysis of different European prevalence studies (including those with selected subgroups) found an overall NAFLD prevalence of 23.7% in 16,735 included subjects[2], corresponding with the prevalence estimate of 22% in this study. NAFLD is less prevalent in Western-Europe when compared to other regions, which show incremental prevalence in North America (24.1%), Asia (27.4%), South America (30.5%) and the Middle East (31.8%)[2]. To date, the Lifelines cohort study with nearly 40,000 participants is the largest study investigating the prevalence of NAFLD in a Western-European cohort. By additionally excluding immigrants, subjects with excessive alcohol use, as well as previously diagnosed hepatitis or cirrhosis, the presently studied cohort is representative in demonstrating a most accurate prevalence figure and coinciding abnormalities in subjects with suspected NAFLD.

When compared to the European prevalence of T2DM and MetS in subjects with suspected NAFLD, prevalence of T2DM was less (9.3% vs. 17.7%) and MetS was more prevalent (54.2% vs. 38.3%) in the Northern region of the Netherlands[2]. This difference could be explained by other studies including subgroup populations, resulting in a combination of different ethnicities and heterogeneity in diagnostic procedures (radiological imaging, ICD codes, self-reported diagnosis and biomarkers) for establishing NAFLD, T2DM and MetS.

All liver enzymes appeared to be increased in the suspected NAFLD group. For ALT, this could be explained by its association with visceral fat, steatosis, inflammation and fibrosis[5]. Remarkably, within the suspected NAFLD group the medians with IQR and means with standard deviations (Table 2) of these liver enzymes were all within the normal reference range used in daily clinical practice. When using the upper limit of normal ALT, 80.3% of subjects in the suspected NAFLD group had normal ALT levels. Others have confirmed these findings. In 79% of subjects with hepatic steatosis[33] and in up to 59% of those with NASH and advanced fibrosis, normal ALT levels were found[34]. This clearly demonstrates the limitations in using ALT levels as a surrogate marker for diagnosing NAFLD and discriminating simple steatosis from steatohepatitis.

A strong association between current smoking, hemoglobin, inflammatory markers (e.g. CRP and total leucocyte count) and suspected NAFLD was found. An association of smoking with NAFLD has not been uniformly reported[5], but may be a confounding environmental stressor. Previous studies have demonstrated that smokers have a higher BMI, increased insulin resistance and that smoking is associated with central fat accumulation, dyslipidemia and concomitant T2DM and MetS, predisposing to comorbidities and risk factors for NAFLD[5,35]. Smoking has been linked to increased hepatic lipid accumulation by modulating the activity of AMPK and SREBP-1, which represent pathways involved in lipid synthesis[36]. The association of a higher hemoglobin level and NAFLD has also been previously demonstrated[37,38] and has been related to progression of NAFLD to NASH and fibrosis[39]. Suggested mechanisms resulting in increased hemoglobin levels are hepatic hypoxia, oxidative stress, formation of reactive oxygen species and lipid peroxidation[37,39]. The association of (subclinical) elevated inflammatory markers and the presence of NAFLD has also been reported in other studies[40]. This may be explained by increased visceral adipose tissue conferring a pro-inflammatory state[41,42]. Also, hepatic free fatty acid oxidation generates oxygen radicals with subsequent lipid peroxidation, cytokine induction and mitochondrial dysfunction, which all conceivably promote inflammation and cause hepatocyte apoptosis and cellular injury. Finally, genetic and gut-derived bacterial factors (in combination with increased intestinal permeability) have an impact on systemic low-grade inflammation[41,43].

The FLI score was used to discriminate between suspected NAFLD and non-NAFLD in this study. The FLI is a well-accepted diagnostic tool for NAFLD, but it is clear that the FLI score is not an absolute measure of hepatic fat accumulation. While histological assessment of liver tissue is still the golden standard for diagnosing NAFLD, liver biopsies have well-known limitations with respect to invasiveness and sampling variability[44] and cannot be performed in very large-scale studies. Alternative, non-invasive strategies for the evaluation of NAFLD are serum biomarkers or the use of imaging techniques. However, imaging techniques are time consuming, expensive and also not feasible in large observational studies. Given these considerations, the recent EASL-EASD-EASO NAFLD guidelines have adopted that serum biomarkers are the preferred diagnostic tool for larger scale screening studies[29]. For the identification of participants with NAFLD in this study, the FLI was used, which was developed from data of the Dionysos Nutrition & Liver Study in Northern Italy[28]. The FLI is one of the three best-validated steatosis biomarkers in the new international accepted guideline[29], has a good steatosis predicting value (AUROC 0.83)[45], and is accurate in detecting NAFLD (accuracy of 0.84 and specificity of 86% for a FLI≥60)[28].

This study is unique in its cohort size of nearly 40,000 participants, which enabled careful calculations on effect sizes, sufficiently powered subgroup analysis and sufficient statistical power to investigate associations. All participants included in the Lifelines Cohort Study have been well examined, with extensive validated questionnaires, standardized anthropometric and laboratory measurements performed in serum samples in one certified laboratory with ditto equipment and quality assessment control for all samples[26]. In addition, included participants in this study had similar distributions of sex, age, BMI, T2DM and MetS compared with the whole Lifelines cohort, so results can be reflected to the total study population. Furthermore, the Lifelines study population has been previously validated, the risk of selection bias is low, is representative and can be generalized to the population of the North of the Netherlands[27].

Several methodological aspects and limitations also need to be addressed. First, this is a cross-sectional study. Thus cause-effect relationships cannot be established with certainty. Second, although the FLI score is an accepted diagnostic tool for NAFLD, it is not an absolute measure of hepatic fat accumulation and thus over- and underestimation of NAFLD could have occurred. Moreover, since the formula of the FLI contains the variables GGT, triglycerides, waist circumference and BMI, the associations of these variables with suspected NAFLD cannot be appropriately ascertained. Finally, since ancestry, alcohol intake, medication use and medical history were based on self-administered questionnaires, misreporting by individuals cannot be excluded. However, considering the large number of subjects, this limitation does not materially affect the interpretation of the presented results.

## Conclusions

In this large study cohort of almost 40,000 subjects performed in the Northern part of the Netherlands, NAFLD is a major suspected health problem. NAFLD is suspected in 22% of a general European population and this group has an increased risk of having T2DM, MetS and a history of cardiovascular disease and impaired renal function. Future analysis of these subjects regarding the development of fibrosis and other population-based studies are mandatory to better understand the natural history of NAFLD and prevent and treat its complications.

## Supporting Information

S1 Table Overview of studies on non-alcoholic fatty liver disease prevalence in Europe. (DOCX)
