# Supplementary material for: Prevalence and determinants of non-alcoholic fatty liver disease in lifelines: A large Dutch population cohort
Source: PLoS One. 2017 Feb 2;12(2):e0171502. doi: 10.1371/journal.pone.0171502 (PMC5289609; doi:10.1371/journal.pone.0171502)
Supplement: S1 Table — (DOCX) [file pone.0171502.s001.docx]

| **Supplementary Table 1. Overview of studies on non-alcoholic fatty liver disease prevalence in Europe.** | | | | | | | |
| --- | --- | --- | --- | --- | --- | --- | --- |
| Author | Publication year | Region | Number of cases | Population | Subjects | Diagnosis method | Prevalence of NAFLD |
| **Armstrong** ^12^ | 2012 | United Kingdom | 1,118 | Subgroup | Adults with incidental abnormal liver function test from primary care centers in Birmingham and Lambeth. | Ultrasonography | 26.4% |
| **Bedogni** ^9^ | 2005 | Italy | 598 | General population | Dionysos study. Age 18-75 years. | Ultrasonography | 22.6% |
| **Bellentani** ^10^ | 2000 | Italy | 257 | Subgroup | Selection from Dionysos study; 67 controls, 66 obese persons, 69 heavy drinkers and 55 obese heavy drinkers. Adults. | Ultrasonography | Controls: 16.4%  Obese: 75.8%  Heavy drinkers: 46.4%  Obese heavy drinkers: 94.5% |
| **Caballeria** ^13^ | 2010 | Spain | 766 | General population | Multicenter; 25 primary healthcare centers. Age 17-83 years. | Ultrasonography | 25.8% |
| **Gastaldelli** ^8^ | 2009 | 14 European countries | 1,307 | General population | 19 Centers. Non-diabetic patients. Age 30-60 years. | Fatty Liver Index > 60 | 17.9% |
| **Kanerva** ^14^ | 2014 | Finland | 1,611 | Subgroup | Participants born in 1943-1944; clinical examination in 2001-2004. | Fatty Liver Index > 60 | 41.2% |
| **Kotronen** ^22^ | 2010 | Finland | 2,766 | Subgroup | Fin-D2D survey. Age 45-74 years. | Blood Test (elevated ALT or AST) | 21.0% |
| **Loguercio** ^15^ | 2001 | Italy | 48 | Subgroup | Adults observed for liver problems at University of Naples who underwent liver biopsy. | Liver biopsy | 29.1% |
| **Ludwig** ^21^ | 2015 | Germany | 1,276 | Subgroup | Adults from the EMIL-study. Cross-sectional survey to analyze thyroid function. | Ultrasonography | 27.4% |
| **Papatheodoridis** ^23^ | 2007 | Greece | 3,063 | Subgroup | Volunteer blood donors. Age 16-66 years. | Blood Test (elevated ALT, AST or GGT) | 17.6% |
| **Pendino** ^24^ | 2005 | Italy | 1,645 | General population (including adolescents) | Population of Cittanova. Age 12-95 years. | Blood Test (elevated ALT, AST, GGT or reduced platelets) | 3.1% |
| **Radu** ^16^ | 2008 | Romania | 3,005 | Subgroup | Adult hospitalized patients for internal and gastrointestinal diseases. | Ultrasonography | 20.0% |
| **Suomela** ^17^ | 2015 | Finland | 1,998 | Subgroup | Population based follow up study. Age 34-49 years. | Ultrasonography | 18.5% |
| **Tarnoki** ^18^ | 2012 | Hungary | 208 | Subgroup | Adult twins. | Ultrasonography | 22.6% |
| **Van der Voort** ^19^ | 2014 | The Netherlands | 2,292 | Subgroup | Population-based cohort study; part of the Rotterdam Study. Age > 55 years. | Ultrasonography | 34.0% |
| **Volzke** ^20^ | 2005 | Germany | 4,222 | General population | Study of Health in Pomerania (SHIP). Age 20-79 years. | Ultrasonography | 29.9% |
| **Zois** ^11^ | 2010 | Greece | 498 | Subgroup | Histological evaluation of tissue samples of autopsies. Age 3-94 year. | Liver biopsy | 31.3% |
| *Fatty Liver Index = (e ^0.953*loge (triglycerides) + 0.139*BMI + 0.718*loge (GGT) + 0.053*waist circumference - 15.745^) / (1 + e ^0.953*loge (triglycerides) + 0.139*BMI + 0.718*loge (GGT) + 0.053*waist circumference - 15.745^) * 100.*  *Abbreviations:* ***ALT****, alanine aminotransferase;* ***AST****, aspartate aminotransferase;* ***GGT****, gamma-glutamyl transferase.* | | | | | | | |
